# Healing the Past by Nurturing the Future: trauma-aware, healing-informed care to improve support for Aboriginal and Torres Strait Islander families – implementation and evaluation study protocol

**DOI:** 10.1136/bmjopen-2024-085555

**Published:** 2024-07-02

**Authors:** Kimberley Ann Jones, Helen Henderson, Tess Bright, Leonie Segal, Olivia Mauerhofer, Katherine Jane Lake, Rebakah Julian, Jhodie Duncan, Anita Raymond, Amanda Jones, Danielle Cameron, Doseena Fergie, Shawana Andrews, Skye Stewart, Caroline Atkinson, Alison Elliot, Belinda Crawford, Janine Mohammed, Gina Bundle, Tanja Hirvonen, Emmanuel Gnanamanickam, Elise Davis, Graham Gee, Helen Herrman, Helen McLachlan, Jane Fisher, Raymond Lovett, Sandy Campbell, Della Anne Forster, Yvonne Clark, Judith Atkinson, Rhonda Marriott, Catherine Chamberlain

**Affiliations:** 1 Indigenous Health Equity Unit, Onemda, Melbourne School of Population and Global Health, The University of Melbourne, Melbourne, Victoria, Australia; 2 Health Economics and Social Policy Group, University of South Australia, Adelaide, South Australia, Australia; 3 Latrobe Regional Hospital, Traralgon, Victoria, Australia; 4 Victoria Aboriginal Child and Community Agency, Melbourne, Victoria, Australia; 5 Poche Centre for Indigenous Health, The University of Melbourne, Melbourne, Victoria, Australia; 6 The Bouverie Centre, La Trobe University, Melbourne, Victoria, Australia; 7 Latrobe City Council, Morwell, Victoria, Australia; 8 Lowitja Institute, Melbourne, Victoria, Australia; 9 Royal Women's Hospital, Melbourne, Victoria, Australia; 10 College of Medicine and Public Health, Flinders University, Adelaide, South Australia, Australia; 11 Adelaide EpiCentre, The University of Adelaide, Adelaide, South Australia, Australia; 12 Murdoch Childrens Research Institute, Melbourne, Victoria, Australia; 13 School of Psychological Sciences, The University of Melbourne, Melbourne, Victoria, Australia; 14 Orygen Youth Health Research Centre and Centre for Youth Mental Health, Orygen Ltd, Melbourne, Victoria, Australia; 15 Centre for Youth Mental Health, The University of Melbourne, Melbourne, Victoria, Australia; 16 Judith Lumley Centre, School of Nursing and Midwifery, LaTrobe University, Melbourne, Victoria, Australia; 17 Global and Women’s Health, Monash University, Melbourne, Victoria, Australia; 18 National Centre for Aboriginal and Torres Strait Islander Wellbeing Research, Australian National University, Canberra, Australian Capital Territory, Australia; 19 Charles Darwin University, Darwin, Northern Territory, Australia; 20 Judith Lumley Centre, La Trobe University, Melbourne, Victoria, Australia; 21 Maternity Services, Royal Women's Hospital, Melbourne, Victoria, Australia; 22 South Australian Health and Medical Research Institute Limited, Adelaide, South Australia, Australia; 23 We Al-li Pty Ltd, Goolmangar, New South Wales, Australia; 24 Ngangk Yira Institute for Change, Murdoch University, Perth, Western Australia, Australia

**Keywords:** health, health equity, implementation science

## Abstract

**Introduction:**

Complex trauma can have serious impacts on the health and well-being of Aboriginal and Torres Strait Islander families. The perinatal period represents a ‘critical window’ for recovery and transforming cycles of trauma into cycles of healing. The Healing the Past by Nurturing the Future (HPNF) project aims to implement and evaluate a programme of strategies to improve support for Aboriginal and Torres Strait islander families experiencing complex trauma.

**Method:**

The HPNF programme was codesigned over 4 years to improve awareness, support, recognition and assessment of trauma. Components include (1) a trauma-aware, healing-informed training and resource package for service providers; (2) trauma-awareness resources for parents; (3) organisational readiness assessment; (4) a database for parents and service providers to identify accessible and appropriate additional support and (5) piloting safe recognition and assessment processes. The programme will be implemented in a large rural health service in Victoria, Australia, over 12 months. Evaluation using a mixed-methods approach will assess feasibility, acceptability, cost, effectiveness and sustainability. This will include service user and provider interviews; service usage and cost auditing; and an administrative linked data study of parent and infant outcomes.

**Analysis:**

Qualitative data will be analysed using reflexive thematic analysis. Quantitative and service usage outcomes will be described as counts and proportions. Evaluation of health outcomes will use interrupted time series analyses. Triangulation of data will be conducted and mapped to the Consolidated Framework for Implementation Research and Reach, Effectiveness, Adoption, Implementation and Maintenance frameworks to understand factors influencing feasibility, acceptability, effectiveness, cost and sustainability.

**Ethics and dissemination:**

Approval granted from St Vincent’s Melbourne Ethics Committee (approval no. 239/22). Data will be disseminated according to the strategy outlined in the codesign study protocol, in-line with the National Health and Medical Research Council Aboriginal and Torres Strait Islander Research Excellence criteria.

**STRENGTHS AND LIMITATIONS OF THIS STUDY**

- An Aboriginal-led, co-designed programme grounded in lived experience that builds capacity for perinatal services to support Aboriginal and Torres Strait Islander families to recover and heal from trauma.
- Implementation of a programme of strategies that promote a whole-of-service behavioural shift in the approach to perinatal care by building the capacity and confidence of service providers to support families in a trauma-aware way.
- A leading example for implementing and evaluating a co-designed programme using Consolidated Framework for Implementation Research and Reach, Effectiveness, Adoption, Implementation and Maintenance frameworks.
- Implementation and evaluation activities have been co-designed in collaboration with the local community and service organisations to strengthen power sharing, impact and acceptability, relevance and operational feasibility.
- All communities and organisations are unique and have different strengths and challenges, accordingly, there may be limits to the generalisability of study results.

## Introduction

 Aboriginal and Torres Strait Islander mothers and babies have poorer health and well-being outcomes than non-Indigenous parents and their children.^1^ Despite initiatives such as Close the Gap, mortality rates of Aboriginal and Torres Strait Islander mothers and their babies remain three and two times higher (respectively) than those in non-Indigenous populations.^2^ The strongest risk factors for perinatal death are birth weight and preterm birth, and babies of Aboriginal and Torres Strait Islander mothers are more likely than non-Indigenous babies to be born too small (9.6% vs 4.7%, respectively) and too early (14% vs 8%).^1^ The gap in rates of children in out-of-home-care (OOHC) has worsened each year, with Aboriginal and Torres Strait Islander children now 10.5 times more likely to be in OOHC than non-Indigenous children nationally, and 22 times more likely in Victoria.^2 3^ The burden of these disparities perpetuates cycles of intergenerational trauma for Aboriginal families. Babies born with low birth weight have over double the odds of Child Protection notifications.^4^ For Aboriginal babies, contact with Child Protection increasingly starts in pregnancy.^5 6^ This leads to Aboriginal mothers avoiding accessing healthcare due to fear of Child Protection involvement,^7^ limiting valuable antenatal care access that could help reduce risks of preterm birth and low birth weight and provide crucial support for other complex needs. Quality perinatal services that safely meet the needs of Aboriginal and Torres Strait Islander parents are clearly needed.

In recent years, there has been increasing focus on the impacts of psychological trauma on access and engagement with services. This has stemmed from observations that people accessing mental health supports are more likely to have been exposed to potentially traumatic events such as witnessing or experiencing actual or threatened death, serious injury or sexual violence^8^ than those experiencing good mental health; that traumatic exposure can lead to significant long-lasting physical, social and emotional health impacts,^9^ and; that traumatic experiences are strikingly prevalent, particularly in childhood.^10^ While the symptoms, aetiology, trajectory and treatment of post-traumatic stress disorder (PTSD) are well understood and researched, complex PTSD (‘complex trauma’) and intergenerational trauma are less so.^11^ Complex trauma is associated with repeated, prolonged, often inescapable and interpersonal traumatic exposures, particularly during childhood and commonly through maltreatment or severe neglect.^12^ In Australia, 62% of people report having experienced at least one type of childhood maltreatment, with 78%–89% of these reporting multiple experiences.^13^ While not all people who experience maltreatment in childhood go on to develop long-term trauma-related difficulties, many do. Complex trauma can be particularly detrimental, impacting people’s ability to experience feelings of safety and trust. People with complex trauma experience the three PTSD symptom clusters of re-experiencing, avoidance and hyperarousal, as well as disturbed relational functioning, emotional dysregulation and negative self-concept,^14^ and they may develop adaptive coping strategies such as dissociation, escapism and suppressed emotions. These challenges can make support-seeking difficult, disempowering and retraumatising.^15^,^18^ The impacts of complex trauma can also impede people’s capacity to nurture and care for their own children^19^ increasing the risk of intergenerational trauma.^20^ Within Aboriginal and Torres Strait Islander contexts, barriers to support-seeking can be exacerbated by institutional racism and fear of Child Protection service involvement fuelled by historic and current high rates of forced child removal,^21^ elevating the risk of retraumatisation during therapeutic engagement.

Prior to colonisation, Aboriginal and Torres Strait Islander peoples experienced robust social and emotional health and well-being fostered by strong connections to land, spirituality, family, community and culture.^22 23^ Invasion and imposition of colonial structures and policies significantly impacted Aboriginal and Torres Strait Islander peoples’ ability to practice this holistic approach to health and well-being.^23 24^ Forced removal of children from family, community and culture perpetuates a legacy of intergenerational trauma through fracturing family relationships, and disrupting connection to culture and kin, contributing to disproportionately poor health outcomes.^25 26^

Pregnancy, birth and early parenthood can be particularly challenging, with an increased risk of trauma-related distress, especially for Aboriginal and Torres Strait Islander parents with complex trauma.^1927^,^29^ This is due to factors such as the intimate nature of pregnancy care, birth, breast feeding and the demands of responding to a baby, which can trigger feelings and responses reminiscent of parents’ own maltreatment or removal^29^ creating high levels of distress. However, pregnancy offers an opportunity to increase support for parents due to regular contact with healthcare professionals.^27 30 31^ This requirement for increased service engagement provides increased opportunities to develop relationships between service providers and families to facilitate culturally safe trauma identification, support and intervention.^27^ The ‘cycle-breaking framework’ for intergenerational trauma proposed by Sperlich *et al*, identifies the perinatal period as an opportune time for transdisciplinary cooperation of health and social services to provide trauma-informed care.^27^ However, evidence shows that perinatal healthcare providers lack the awareness, skills and knowledge to provide trauma-informed care.^32^ An Australia-wide survey of primary maternity care providers found 43% were ‘not satisfied’ with the ability of their service to address trauma.^33^ A study of 126 child welfare, juvenile justice, mental health and education service providers found they had a good understanding of what trauma-informed practice is, and its importance, but most lacked skills and strategies to support people with trauma experiences.^34^ Service providers expressed a need for skills-based training in how to work with families exposed to trauma, better trauma-informed assessment tools and support for linking families to the most appropriate treatment.^34^ To transform cycles of intergenerational trauma, such training should incorporate Indigenous knowledges^35^ and ensure trauma-informed support is provided in a culturally safe and appropriate way. Service providers need mentorship or supervision as questions arise. Further, evaluation of trauma-informed programmes using an Indigenous lens is essential.

Through the provision of culturally safe, trauma-aware perinatal care we can intervene in cycles of intergenerational trauma to promote cycles of healing and nurturing and begin to close the gap in maternal and child health and well-being outcomes for Aboriginal and Torres Strait Islander parents.

## Aims and objectives

Healing the Past by Nurturing the Future (HPNF) is an Aboriginal-led, community-based participatory action research project that aims to transform cycles of complex and intergenerational trauma into cycles of healing for Aboriginal and Torres Strait Islander families. The project will implement a programme of strategies that promote a whole-of-service behavioural shift in the approach to perinatal care by building the capacity and confidence of service providers to support families in a trauma-aware way. The HPNF programme is designed to raise awareness of trauma and its impacts and equip service providers with the skills, knowledge and resources needed to provide culturally safe, trauma-aware, healing-informed perinatal care.

Through the implementation of trauma-aware, healing-informed perinatal care HPNF will answer the following research questions:

- What are the experiences of Aboriginal and Torres Strait Islander parents receiving trauma-aware, healing-informed perinatal care (acceptability)?
- What are the barriers and enablers to implementation (feasibility)?
- What are the costs of implementing trauma-aware, healing-informed perinatal care compared with usual care (cost-effectiveness)?
- What is the impact on health and socioemotional outcomes for Aboriginal and Torres Strait Islander parents and their infant children (effectiveness)?

The development of the HPNF programme and conceptual framework are described in detail elsewhere.^19^

## Methods and analysis

**Figure 1:**
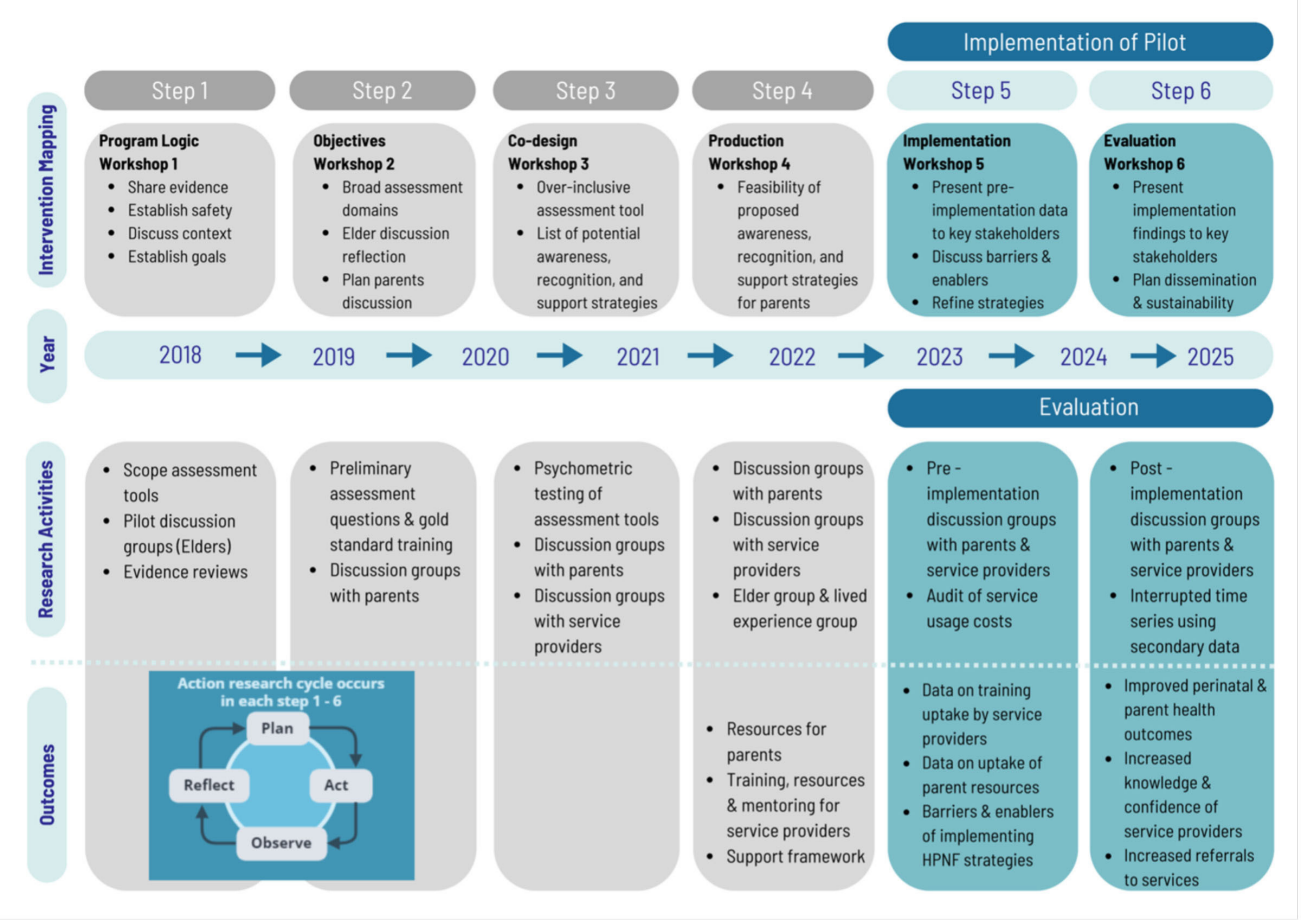
The Healing the Past by Nurturing the Future intervention mapping research plan.

The HPNF research plan (figure 1) illustrates an intervention mapping process describing formative research steps 1–4 during which the HPNF programme was developed. Steps 5–6 represent the implementation and evaluation steps described in the current paper (November 2023–December 2025). Participatory action research cycles are illustrated to describe the process of planning, action, observation and reflection that are built into the research plan at each step.

### Patient and public involvement

The HPNF programme was developed and designed through a rigorous 4-year co-design process described in previous publications.^19 28 36 37^

The HPNF project is being conducted using community-based participatory action research principles. Participative processes are embedded in the research design (e.g., workshops and discussion groups). Preimplementation data will inform the development of resources and refinement of implementation. Implementation workshops will serve to collect feedback during implementation to further refine he HPNF programme to meet the needs of the patients and providers at the implementation site. Data generated will be shared according to the dissemination strategy outlined in the published co-design phase study protocol.^19^

### Conceptual framework

**Figure 2:**
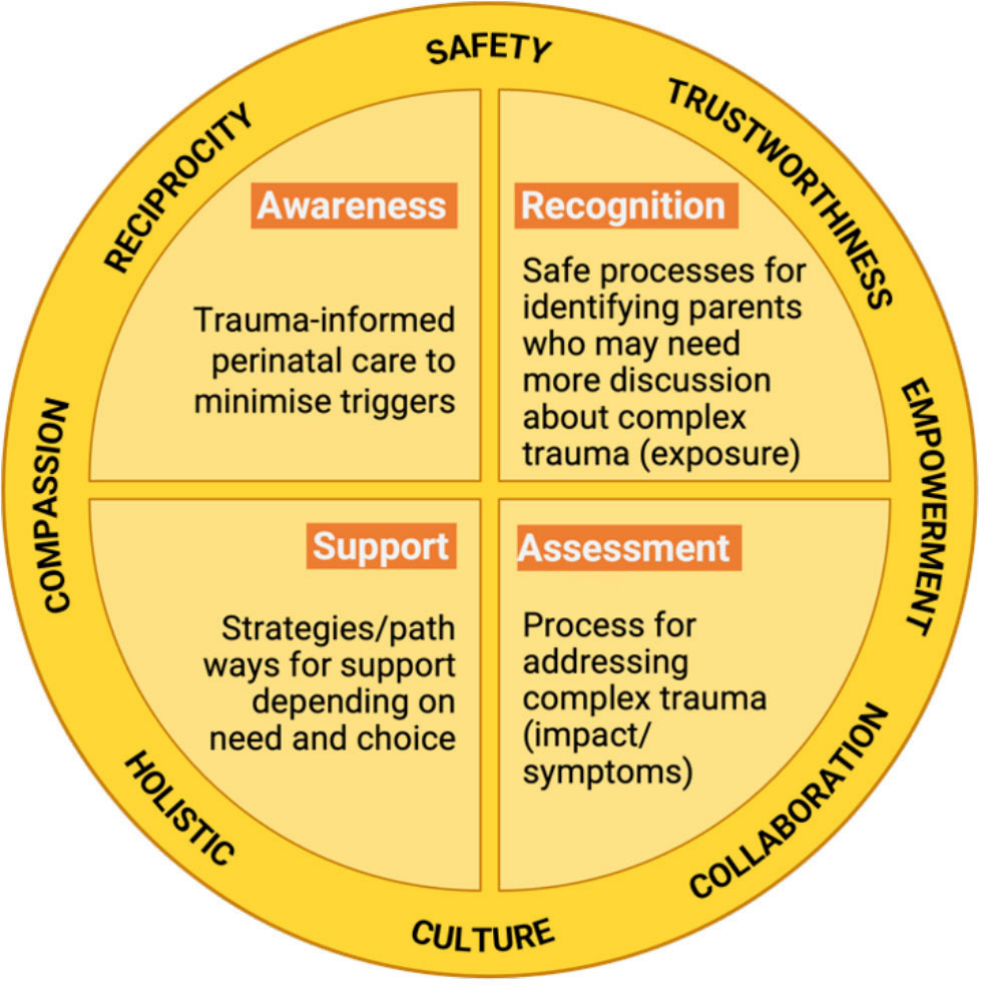
The Healing the Past by Nurturing the Future project conceptual framework.

The HPNF conceptual framework^19^ was designed and used in steps 1–4 of the development of the HPNF programme. It presents eight essential values for trauma-aware, healing-informed care: safety, trustworthiness, empowerment, collaboration, culture, holistic, compassion and reciprocity, supporting four primary domains: awareness, recognition, assessment and support (figure 2). The HPNF programme strategies address each primary domain while the values are used to guide implementation.

### Intervention strategies aligning with HPNF domains

#### Awareness and recognition

##### HPNF training programme

To deliver trauma-aware, healing-informed perinatal care for Aboriginal and Torres Strait Islander families, service providers must be aware of intergenerational trauma, its causes and triggers. Furthermore, service providers must be able to recognise when parents may be experiencing trauma responses or symptoms and respond in ways that ensure parent safety and support, avoiding retraumatisation. To build service provider awareness of intergenerational trauma, a comprehensive training package was developed including introductory, intermediate and advanced training courses. All staff working in a role that has any contact with Aboriginal and Torres Strait Islander parents, or managing roles that do, will be invited to complete the training package, recognising that both clinical and non-clinical staff across the health service need to adopt a trauma-aware approach to improve safety and minimise the risk of retraumatisation.

##### Training package courses

Introductory: a modularised online course developed in collaboration with project partners Emerging Minds. This 5-hour course provides foundational knowledge for service providers covering Aboriginal and Torres Strait Islander history, impacts of colonisation and trauma.^38^

Intermediate: an intensive 1-day, in-person training course that aims to build service providers’ practical skills in the delivery of trauma-aware, healing-informed care. Course content includes the neurobiology of trauma, how to recognise when an individual is experiencing trauma symptoms and responses and how to use a trauma-aware approach to care for Aboriginal and Torres Strait Islander parents during the perinatal period that avoids retraumatising and inspires hope and healing. Intermediate training will be delivered initially by members of the HPNF research team and handed on to hospital education staff to ensure long-term sustainability. Each session will be cofacilitated by Aboriginal and non-Aboriginal content experts to foster a ‘two-way learning’ experience whereby attendees gain content from both mainstream perspectives and through an Indigenous lens.

Advanced: a training course of 2×2 days for service providers who wish to further extend their knowledge and skills in trauma-informed care. Days 1 and 2 provide a deeper understanding of trauma-aware healing-informed care. Days 3 and 4 focus on building a community of practice within the service system. Service providers who complete the advanced training will become project ‘champions’ in their workplace promoting trauma-aware, healing-informed care. They will be provided mentorship for 12 months. The advanced training is designed to embed trauma-aware healing-informed practice into the workplace and ensure sustainability of the strategies long term. Advanced training will be delivered by project partners We Al-li. We Al-li workshops integrate Indigenous cultural processes of education, conflict management and personal/social healing with Eastern and Western therapeutic skills for trauma recovery using an experiential learning model.

##### Parent resources

The research team will work with local Aboriginal and Torres Strait Islander parents and community organisations to develop a suite of resources for parents tailored to local needs. These resources aim to improve parents’ awareness of trauma and its impacts and highlight strength and resilience. Resources may include a dad’s pack with resources specifically designed for supporting fathers during the perinatal period; a storybook or other creative output developed with the local community; and a parent booklet drawing on Aboriginal and Torres Strait Islander principles of child-rearing.

### Support

#### Support database

The HPNF Support Database will provide an online directory of services for Aboriginal and Torres Strait Islander parents including detailed information about accessibility. The aim of the Support Database is to ensure parents have available to them a wide range of accessible, holistic supports to meet complex needs and to identify and highlight service gaps. The HPNF research team will work with unit managers and community organisations to collect service information to develop the database. Resources that have been designed specifically for Aboriginal parents or Aboriginal people experiencing trauma will also be available in an online resource repository being developed as part of an associated project (Replanting the Birthing Trees). These will include websites, helplines, brochures and any other resources deemed useful, accetable and appropraite by a group of Aboriginal service users and providers. Resources will be sourced through a grey literature search and local networks.

### Assessment

#### Safe assessment tools

The HPNF project will assess the feasibility and acceptability of the Aboriginal and Torres Strait Islander Complex Trauma and Strengths Questionnaire (ACTSQ) assessment tool for complex trauma for Aboriginal and Torres Strait Islander parents.^39^ Incorporating Aboriginal and Torres Strait Islander concepts of social and emotional well-being, this tool will enable culturally grounded assessment for primary healthcare planning for Aboriginal and Torres Strait Islander families.

### Research project setting

Implementation will take place in Gunaikurnai country in Inner Gippsland (Victoria, Australia). The Latrobe local government area has a Socio-Economic Indexes for Areas Quintile of 1, indicating a high level of disadvantage in the area.^40^ The implementation site is a ‘level 5’ capability health service (primary and specialised maternity services for women with normal-to-moderate risk pregnancies) that provides services to a large rural area and is the main provider of mental health services for the region. The HPNF project is being conducted in partnership with Latrobe Regional Health (LRH), the community-based Victorian Aboriginal Child and Community Agency (VACCA) and Latrobe City Council. VACCA and Victoria Aboriginal Community Controlled Health Organization Koori Maternity Service (VACCHO KMS) offer support for 70–100 Aboriginal and/or Torres Strait Islander parents (~50 births) a year across the Gippsland catchment area.

### Governance

In line with the participatory action research approach, it is imperative that the HPNF project builds strong relationships within the implementation site and local community services to ensure the uptake and ongoing sustainability of the HPNF programme. A site implementation team (SIT) has been established to act as project champions, foster local ownership and community participation, identify and incorporate relevant practice-based evidence and resources, address structural barriers and oversee local adaptations. Members of the SIT include HPNF research team members and research managers, Aboriginal Liaison Officers, VACCA representatives, educators, social workers, maternity nurse unit managers and executive team members from the implementation site. The HPNF project receives governance from its Aboriginal-led investigator group of Aboriginal and Torres Strait Islander and non-Indigenous researchers. Strategies and interventions implemented will also be shared with the Consumer Advisory Committee at the implementation site which also has an Aboriginal consumer representation, in support of improving consumer experience and engagement.

### Participants

#### Parents

##### Eligibility

Interviews/discussion groups: Aboriginal and Torres Strait Islander parents aged 16 years or above with a child under 5 years born at the implementation site.

Maternal well-being study: Aboriginal and Torres Strait Islander parents aged 16 years or above with a baby aged 3–9 months that was born at the implementation site.

##### Interview/discussion groups

Interviews and/or group discussions (yarning groups) with 15–20 participants will be conducted 2–3 months preimplementation and 2–3 months postimplementation to explore parents’ experiences of perinatal care at the implementation site. Postimplementation groups will also include a question about the HPNF resources they received.

A yarning/discussion group will be conducted with a group of local fathers to understand what resources would be useful for new dads in the perinatal period. These resources will be developed and distributed.

##### Separate sample pre–post implementation maternal well-being study

Parents participating in the parent preimplementation or postimplementation interview/discussion groups, who have a child aged 3–9 months born at the implementation site will be invited to participate in an additional interview where they will complete the ACTSQ; Kessler Psychological Distress Scale (K5); and parenting self-efficacy (Tool to Measure Parenting Self-Efficacy).^39 41 42^

##### Interrupted time series

An interrupted time series study will use routinely collected perinatal, maternal and child health data, and child protection services, emergency department and hospital separations data on children (and their mothers) born 2 years prior to and 2 years after implementation, to evaluate health service use, maternal and child health outcomes, child protection notifications and out of home care placements and feasibility for future large-scale implementation of the project. Data from another regional local government area will serve as a comparison site for additional comparative analyses and will be selected based on similar demographics: per cent Aboriginal and/or Torres Strait Islander, age, socioeconomic status, education level and key maternal and child health indicators using data from the preimplementation period.

Approximately 40–50 Aboriginal and/or Torres Strait Islander women give birth at the implementation site each year. Therefore, the whole-of-population interrupted time series analyses will include approximately 200 women who have given birth over the study period from January 2021 to December 2026.

### Service providers

#### Eligibility

Service providers who work in the implementation site region in a role associated with the delivery of perinatal care, or who hold management or executive level roles that have oversight of these roles (eg, midwifery, nursing, obstetrics and gynaecology, allied health, educators, administrative staff).

#### Interviews

Service providers will be invited to participate in interviews 2–3 months preimplementation and 2–3 months postimplementation.

Service provider interviews will include semistructured interview questions about their perceptions of care provided to Aboriginal and Torres Strait Islander parents who access perinatal care services at the implementation site and how well-equipped and supported they feel to provide good quality care.

Service providers participating in interviews will also be asked to complete a Barriers and Enablers to Trauma-Informed Care Implementation (BETICI) measure.^43^ The BETICI survey seeks to understand service providers’ perspectives about barriers and enablers to using a trauma-informed approach to care in their workplace. Results will be used to inform service provider training development.

Postimplementation interview/discussion group participants will be asked the same set of questions as the preimplementation cohort, with additional questions relating to barriers and enablers encountered in implementation.

#### Service provider feedback portal

All service providers who attend HPNF training will have access to an online feedback portal to provide feedback during the implementation period. The portal will ask questions related to Consolidated Framework for Implementation Research (CFIR) constructs on a 5-point Likert scale and open-ended questions to provide reflections on aspects of implementation. The aim of the portal is to ascertain service provider satisfaction with the intervention implementation and service provider training, and barriers or enablers in implementing skills learnt in the training. Providers will be asked to indicate their interest in using the portal during registration for HPNF training. Those indicating interest will receive periodic emails encouraging entry of feedback.

#### Knowledge, attitudes and practices

Service providers will be asked to self-complete a knowledge, attitudes and practices (KAP) survey via REDCap^43^ before and after the HPNF training. An email will be sent to participants 3 months after they complete their training inviting them to complete the KAP again as a follow-up survey. The KAP tool was developed in the initial codesign phase of the HPNF project to assess: (1) what provider know and understand about a topic (knowledge domain); (2) what providers think or feel about the topic (attitudes domain) and (3) what providers do in practice, how they demonstrate or have capacity (self-efficacy) to apply their knowledge and attitudes (practices domain).^43^

#### Organisational readiness assessment

An organisational readiness assessment using an adapted Organisational Readiness for Implementing Change (ORIC) survey^44^ will be conducted at the implementation site to establish the willingness and capacity for change in the organisation prior to implementation, to help inform the implementation strategies and content of training. Distribution of the survey will occur via email from the implementation site executive team and all staff will be invited to complete the survey via a link to an online REDCap survey.

#### Policy review

The HPNF research team will conduct a review of current policies at the implementation site to understand current policy directives that impact the care provided to Aboriginal and Torres Strait Islander parents. This information will be used to develop a plan with the champion group where it is determined there is potential capacity for the project to impact policy change to improve support for Aboriginal and Torres Strait Islander parents.

### Implementation team

#### SIT journaling

An online ‘journal’ will be established and SIT members will be encouraged to regularly record their reflections on implementation progress including barriers and facilitators to implementation. Each SIT meeting will include a standing agenda item for members to verbally report reflections on implementation progress barriers and enablers—these data will be added to the SIT journal by a member of the research team.

#### Key stakeholder workshops

Approximately 50 key stakeholders from the local area (service providers, researchers, policy-makers, representatives from executive, education, research and ward managers and staff and community leaders) will be invited to participate in a workshop to codesign solutions to barriers highlighted in the implementation period and develop a sustainability plan to embed changes into the health service. Workshop 5 will be conducted at the end of the implementation period. Workshop 6 will be conducted at the conclusion of postimplementation data collection and analysis. This postimplementation workshop will present overall project results, update and assess progress in solutions to barriers highlighted in workshop 5, and finalise planning for embedding programme strategies into the health service to ensure sustainability and maintenance of any gains beyond the life of the project.

### Recruitment

Parents will be recruited for interviews/discussion groups and the separate samples pre–post test maternal well-being study through flyers and word-of-mouth facilitated by project partners at key local community organisations who have established relationships with local Aboriginal and Torres Strait Islander parent groups.

Service providers will be recruited for interviews, organisational readiness assessment, BETICI survey, KAP surveys, feedback portal and training via research team-delivered information sessions, email invitation and flyer and newsletter promotion at the implementation site.

### Consent

Informed consent will be obtained from all participants for all data collection apart from deidentified secondary data where a waiver has been approved as, given the nature of the datasets, it is impracticable to seek consent from the participants. The secondary data from administrative datasets will be securely shared in a deidentified form.

## Research design

The CFIR will guide the design and evaluation of the implementation methods.^45^

The CFIR model will be applied using a codesign approach ensuring that implementation strategies adequately address contextual factors.

### Implementation

The HPNF project is employing a participatory action research approach. As such, incremental changes to the programme are expected over time to incorporate improvements and modifications to address gaps identified through observe, reflect plan, act cycles (figure 1). Implementation activities are provided in table 1.

**Table 1:** HPNF programme intervention strategies

Intervention component	Description
Service providers	
HPNF info sessions	Information session series early in preimplementation period to explain the project to service providers
Hospital commitment	Mandate of intermediate training—paid study leave for service providers to attend 1-day training
Site visits	Regular research team visits to build relationships and foster collaboration
Ongoing site participation	Fortnightly SIT meetings
Service provider education package	Introductory—online self-paced total 5 hours
	Intermediate—in-person, 1 day
	Advanced—2×2 day in person
HPNF action group	Action group guide, mentoring and regular meetings
Champions	Advanced HPNF 4-day training, champions guide, mentoring
Online website resources	Resource repository—database of trauma-related resources including brochures, websites
	Support services directory—database of support services for parents including accessibility information
Feedback portal	Online portal with specific yes/no barriers and enablers questions and free text sections for providers to feedback on implementation progress
Posters/reminders	Clinical visual tools—developed throughout implementation using feedback from providers in feedback portal, interviews and evaluation
Parents	
Parent resource	Creative activity where Aboriginal and Torres Strait Islander parents will lead the design of a resource or activity to express and explore perinatal experiences
Dad’s pack	Physical pack designed in community with Aboriginal and Torres Strait Islander dads with practical resources and information for support
Parenting booklet	Community-designed booklet drawing on Aboriginal and Torres Strait Islander knowledges to raise parent’s awareness and understanding about trauma

HPNFHealing the Past by Nurturing the FutureSITsite implementation team

The CFIR has been used to assist with planning and organising the implementation and evaluation process. Online supplemental file 1 outlines how each CFIR construct will be used to measure progress and identify barriers and enablers across important aspects throughout the implementation and evaluation process.

### Evaluation

In addition to evaluation constructs within the CFIR, a mixed-method approach will be employed using the RE-AIM framework^46^ to evaluate the reach, effectiveness, adoption, implementation and maintenance of the HPNF programme. The RE-AIM framework is designed to evaluate the impact and sustainability of interventions and encourage consideration of both internal and external validity. Details of the RE-AIM evaluation activities are presented in online supplemental file 2.

## Ethics and dissemination

### Ethics

The implementation and evaluation research methods have been developed in line with the National Health and Medical Research Council (NHMRC) guidelines for ethical conduct in Aboriginal and Torres Strait Islander Health Research. Ethics approval has been received from St Vincent’s Ethics Committee (approval no. 239/22), with site governance from LRH (2023-36 SSA). All participant data collection and research activities have informed consent procedures that have been reviewed and approved by the above ethics committee.

### Dissemination

As described above, the HPNF project will adopt a community-based participatory action research approach, embedding participative processes are all aspects of the research process including dissemination of data generated. The dissemination strategy was developed to align with the NHMRC Aboriginal and Torres Strait Islander Research Excellence criteria (table 2).

**Table 2:** HPNF dissemination activities

Dissemination activity	Stakeholders
Opportunity to comment on draft findings and provision of final findings in academic and plain language format.	All research participants
Quarterly newsletter updates with project highlights and links to further information	All project investigators and partners, mailing list recipients
A designated project website providing access to all project reports, plain language summaries, presentations and quarterly newsletters	Community
Presentations offered at community meetings incorporating two-way information exchange	Partner organisation staff
Presentations at national and international conferences and Aboriginal and Torres Strait Islander research forums	Community
All publications are in open-access format with links available on the Lowitja Institute website and project website.	Community
Use of art, presentations and other culturally relevant mediums to share information	Community

HPNFHealing the Past by Nurturing the Future

## Discussion

The HPNF project will demonstrate the implementation of community co-designed strategies that aim to improve the support that Aboriginal and Torres Strait Islander families receive during the perinatal period, and maternal and child health and well-being outcomes. The HPNF programme centres on raising awareness of trauma and its impacts, to reduce the risk of retraumatisation during the provision of perinatal care and to create an environment where healing can be facilitated. The development of a service and resource database will also improve supports for families experiencing trauma. Additionally, piloting of the ACTSQ will contribute to safer assessment of trauma. Evaluation of the HPNF effectiveness, adoption, implementation and maintenance will inform scale-up of the programme. The HPNF project has the potential to achieve organisational level behaviour change that will improve the safety and accessibility of support for Aboriginal and Torres Strait Islander families. This change has a strong potential to impact the high rates of child removal and so contribute to Closing the Gap between Indigenous and non-Indigenous children living in OOHC. Through improving support in the critical perinatal period, providers can begin to support families to stay together and transform cycles of trauma into cycles of healing.

## supplementary material

10.1136/bmjopen-2024-085555 online supplemental file 1

10.1136/bmjopen-2024-085555 online supplemental file 2

## References

[1] Australian Institute of Health and Welfare (2023). Aboriginal and Torres Strait Islander mothers and babies Canberra Australian Government.

[2] Australian Government (2023). Closing the Gap Information Repository Canberra Productivity Commission.

[3] Productivity Commission (2023). Closing the gap annual data compilation report Canberra Australian government.

[4] He V, Guthridge S, Leckning B (2019). From Birth to Five: A Multiagency Data-Linkage Study to Inform A Public Health Response to Child Protection in the Northern Territory.

[5] O’Donnell M, Lima F, Maclean M (2023). Infant and pre-birth involvement with child protection across Australia. Child Maltreat.

[6] Wise S, Corrales T (2023). Discussion of the Knowns and unknowns of child protection during pregnancy in Australia. *Australian Social Work*.

[7] Davis M (2019). Family is culture: independent review of aboriginal children and young people in out of home care in New South Wales. Sydney, NSW.

[8] American Psychiatric Association DSM-V. Diagnostic and Statistical Manual of Mental Disorders.

[9] Dudgeon P, Watson M, Holland C (2017). Trauma in the aboriginal and Torres Strait Islander population. Australian Clinical Psychologist.

[10] Stoltenborgh M, Bakermans‐Kranenburg MJ, Alink LRA (2015). The prevalence of child Maltreatment across the globe: review of a series of Meta‐Analyses. *Child Abuse Review*.

[11] Alexander PC (2015). Intergenerational Cycles of Trauma and Violence.

[12] Singh J, Prakash J, Yadav P (2021). Complex psychological trauma. Ind Psychiatry J.

[13] Haslam D, Mathews B, Pacella R (2023). The prevalence and impact of child Maltreatment in Australia: findings from the Australian child Maltreatment study (report no.: 0645772801). Brief Report.

[14] Cloitre M, Garvert DW, Weiss B (2014). Distinguishing PTSD, complex PTSD, and borderline personality disorder: A latent class analysis. *Eur J Psychotraumatol*.

[15] Littleton H, Horsley S, John S (2007). Trauma coping strategies and psychological distress: a Meta‐Analysis. J Trauma Stress.

[16] Curran E, Perra O, Rosato M (2021). Complex childhood trauma, gender and depression: patterns and correlates of help-seeking and maladaptive coping. J Affect Disord.

[17] Kerig PK (2019). Linking childhood trauma exposure to adolescent justice involvement: the concept of Posttraumatic Risk‐Seeking. Clin Psychol: Sci Pract.

[18] Sweeney A, Taggart D (2018). (Mis)Understanding trauma-informed approaches in mental health. J Ment Health.

[19] Chamberlain C, Gee G, Brown SJ (2019). Healing the past by Nurturing the future—Co-designing perinatal strategies for aboriginal and Torres Strait Islander parents experiencing complex trauma: framework and protocol for a community-based Participatory action research study. BMJ Open.

[20] Reese EM, Barlow MJ, Dillon M (2022). Intergenerational transmission of trauma: the mediating effects of family health. Int J Environ Res Public Health.

[21] Markwick A, Ansari Z, Clinch D (2019). Experiences of racism among aboriginal and Torres Strait Islander adults living in the Australian state of Victoria: a cross-sectional population-based study. BMC Public Health.

[22] Lovett R (2014). A history of health services for aboriginal and Torres Strait Islander people. Yatdjuligin: aboriginal and Torres Strait Islander nursing and Midwifery care.

[23] Gee G, Dudgeon P, Schultz C (2014). Aboriginal and Torres Strait Islander social and emotional wellbeing. working together: aboriginal and Torres Strait Islander mental health and wellbeing principles and practice.

[24] Sherwood J (2013). Colonisation–it’s bad for your health: the context of aboriginal health. Contemporary Nurse.

[25] Zubrick S, Silburn S, Lawrence D (2005). The Western Australian Aboriginal Child Health Survey: Forced Separation from Natural Family, Forced Relocation from Traditional Country or Homeland, and Social and Emotional Wellbeing of Aboriginal Children and Young People.

[26] O’Donnell M, Taplin S, Marriott R (2019). Infant removals: the need to address the over-representation of aboriginal infants and community concerns of another ‘stolen generation. Child Abuse Neglect.

[27] Sperlich M, Seng J, Rowe H (2017). A cycles-breaking framework to disrupt Intergenerational patterns of Maltreatment and vulnerability during the childbearing year. J Obstet Gynecol Neonatal Nurs.

[28] Chamberlain C, Clark Y, Hokke S (2021). Healing the past by Nurturing the future: aboriginal parents’ views of what helps support recovery from complex trauma: indigenous health and well-being: targeted primary health care across the life course. Primary Health Care Res Develop.

[29] Muzik M, Rosenblum K (2018). Motherhood in the Face of Trauma.

[30] Fava NM, Simon VA, Smith E (2016). Perceptions of general and parenting-specific Posttraumatic change among postpartum mothers with histories of childhood Maltreatment. Child Abuse Negl.

[31] Green-Miller SN (2012). Intergenerational parenting experiences and implications for effective interventions of women in recovery: Walden University.

[32] Fiolet R, Roberts V, Bloomer MJ (2021). Trauma-informed care: why is it so important in primary health care. Collegian.

[33] Highet NJ, Goddard AK (2014). Aboriginal Perinatal Mental Health Mapping Project.

[34] Donisch K, Bray C, Gewirtz A (2016). Child welfare, juvenile justice, mental health, and education providers’ Conceptualizations of trauma-informed practice. Child Maltreat.

[35] Marriott R, Reibel T (2021). Resilience, renewal and hope in Australian indigenous-led primary health care initiatives. *Prim Health Care Res Dev*.

[36] Clark Y, Gee G, Ralph N (2020). The healing the past by Nurturing the future: cultural and emotional safety framework. J Indig Wellbeing.

[37] Ralph N, Clark Y, Gee G (2018). Healing the past by Nurturing the future: perinatal support for aboriginal and Torres Strait Islander parents who have experienced complex childhood trauma-workshop one report.

[38] Emerging Minds Learning (2023). Healing the Past by Nurturing the Future: Working with Aboriginal and Torres Strait Islander Families.

[39] Gee G, Bright T, Morgan A (2024). Aboriginal and Torres Strait Islander complex trauma and strengths questionnaire: Psychometric evaluation. Austral J Psychol.

[40] Australian Bureau of Statistics (2023). Census of Population and Housing: Socio-Economic Indexes for Areas (SEIFA), Australia, 2021.

[41] Bloomfield L, Kendall S (2007). Testing a parenting programme evaluation tool as a Pre‐And Post‐Course measure of parenting Self‐Efficacy. J Adv Nurs.

[42] Department of Health (2018). Primary Mental Health Care Minimum Data Set Scoring the Kessler 5.

[43] Reid C, Bennetts SK, Nicholson JM (2023). Rural primary care workforce views on Trauma‐Informed care for parents experiencing complex trauma: A descriptive study. Aust J Rural Health.

[44] Shea CM, Jacobs SR, Esserman DA (2014). Organizational readiness for implementing change: a Psychometric assessment of a new measure. Implementation Sci.

[45] Damschroder LJ, Aron DC, Keith RE (2009). Fostering implementation of health services research findings into practice: a consolidated framework for advancing implementation science. Implement Sci.

[46] Glasgow RE, Vogt TM, Boles SM (1999). Evaluating the public health impact of health promotion interventions: the RE-AIM framework. Am J Public Health.

