# Supplementary material for: Healing the Past by Nurturing the Future: trauma-aware, healing-informed care to improve support for Aboriginal and Torres Strait Islander families – implementation and evaluation study protocol
Source: BMJ Open. 2024 Jul 2;14(7):e085555. doi: 10.1136/bmjopen-2024-085555 (PMC11227778; doi:10.1136/bmjopen-2024-085555)
Supplement: online supplemental file 1 [file bmjopen-2024-085555-s001.pdf]

Supplementary file 1

Consolidated Framework of Implementation Research (CFIR) application

CFIR implementation		
**CFIR construct**	**Description**	**Application/assessment**
**Intervention characteristics**		
**Intervention Source**	Perception of key stakeholders about whether the intervention is externally or internally developed.	- It will be explained in staff information sessions and in-person training how the four-year co-design phase of the project informed the development of the intervention.<br>- It will be emphasized in the training how the data collected from service providers informed development.
**Evidence Strength & quality**	The extent to which service providers perceive the evidence supporting the intervention as valid and of sufficient quality to result in positive outcomes	- KAP survey results will highlight service provider perceptions and inform training content adaptations.<br>- KAP: *There is evidence that trauma-informed approaches improve outcomes for families*
**Relative Advantage**	Relative advantage – the extent to which service providers perceive the HPNF intervention as more advantageous than usual care	- KAP and BETICI survey questions will highlight service provider perceptions and where barriers are identified, training content will be adjusted.<br>- KAP: *Using a trauma-informed approach means parents get better* care.<br>- BETICI: *Trauma-informed approaches would/do greatly improve outcomes for all parents.*
**Adaptability**	Can the intervention be adapted or tailored to fit the needs of the implementation site?	- Implementation strategies will be co-developed, regularly reviewed and adapted with SIT team and HPNF action group to adapt to local setting.<br>- Service provider feedback portal will collect user feedback in real time which will be discussed with SIT and action groups to ensure barriers are managed
**Complexity**	The perception of service providers regarding how complex the intervention is to implement	- Service provider training will provide skills and confidence to implement trauma-informed practice.<br>- Service providers will be asked to share perceived barriers to the strategies, and these will be workshopped in training sessions, with SIT, HPNF action group and at Workshop 5<br>- KAP: *Adopting a trauma-informed approach in my job is too hard.*

**Design Quality and Packaging**	Perceived excellence in how the intervention is bundled, presented, and assembled.	- Service providers will be asked to complete a set of evaluation questions at the end of each training level assessing their perceptions of training quality, accessibility etc. The service provider feedback portal will also provide an opportunity for service providers to comment on the usefulness and applicability of the training. Responses to these questions will inform any required adaptations to content and delivery of the training.
**Cost**	how much will it cost to implement the intervention?	- A cost analysis will be conducted
**Outer setting**		
**Patient Needs & Resources**	The extent to which patient needs, as well as barriers and facilitators to meet those needs are accurately known and prioritised by the organisation.	- Qualitative data from local parent interviews and focus groups will be fed back to providers in intermediate training sessions. This data will highlight parent's perceptions are care and barriers and facilitators to safer more accessible care. These findings will inform training content.<br>- Service provider interviews will highlight perceptions of the quality of care provided to Aboriginal parents, and potential improvements.<br>- BETICI:<br>*Trauma-informed approaches are not necessary for the parents who access our services.*<br>*Trauma-informed approaches would/do greatly improve outcomes for all parents.*<br>- KAP:<br>*Aboriginal and Torres Strait Islander families often find healthcare settings particularly distressing.*<br>*I am able to create safe and welcoming spaces for people impacted by trauma.*<br>*I take the time to build trusting relationships with families.*
**Cosmopolitanism**	The degree to which an organization is networked with other external organizations.	- Service providers will be asked in interviews about their perceptions of how well organisations and services work collaboratively together to ensure families get good quality care.<br>- KAP:<br>*I collaborate with internal and external service providers when working with Aboriginal and Torres Strait Islander families.*<br>*I can name three local services for Aboriginal and Torres Strait Islander families.*<br>- FEEDBACK PORTAL:

		My service has strong collaborations and relationships with other organisations and services to support families
**External Policy & Incentives**	A broad construct that includes external strategies to spread interventions including policy and regulations (governmental or other central entity), external mandates, recommendations and guidelines, pay-for-performance, collaboratives, and public or benchmark reporting.	- The SIT will collect information about the extent to which external policies, guidelines, and incentives impact implementation through conduct of a policy review.<br>- Training will incorporate content on how the intervention can help progress towards Closing the Gap targets.<br>- KAP:<br>*Closing the Gap targets 2 and 12 are on track to being achieved on time (reverse scored)*<br>- The HPNF action group will be encouraged to develop strategies to highlight and track progress towards Closing the Gap targets
**Inner setting**		
**Structural Characteristics**	The social architecture, age, maturity, and size of an organization.	- The SIT will review potential and actual barriers to implementation and workshop solutions at each fortnightly meeting.<br>- Service providers will be encouraged to use the feedback portal to communicate perceived barriers to implementation. These responses will be discussed at each SIT meeting.<br>- Organisational readiness survey results will be used to ascertain information about the characteristics of the organisation's workforce that may affect implementation.<br>BETICI:<br>- *Trauma-informed approaches would be/are considered a burden on resources.*<br>Questions relating to subconstructs:<br>**Physical infrastructure:**<br>- FEEDBACK PORTAL: *The physical infrastructure within my workplace* [barrier/enabler to using a trauma-informed approach]<br>- KAP*: I am able to create safe and welcoming spaces.*<br>**Policy and procedures:**<br>- FEEDBACK PORTAL: *Policies and/or procedures at my workplace* [barrier/enabler]<br>**Workforce:**<br>- FEEDBACK PORTAL: *The number of staff we have* [barrier/enabler]

**Networks and Communications**	The nature and quality of webs of social networks and the nature and quality of formal and informal communications within an organization.	- Service provider interviews: providers will be asked what improvements are needed and what is working well regarding the care provided at their service, as well as how well-equipped they feel to provide care to Aboriginal parents.<br>- Data from service provider interviews will be discussed in SIT meetings and will inform implementation activities.<br><br>Questions relating to subconstructs:<br>**Relatedness:** service providers will respond to questions in organisational readiness survey related to their beliefs about the level of commitment, motivation, and desire to implement changes.<br>**Organisation level communication:**<br>- FEEDBACK PORTAL: *Communication across my whole organisation* [barrier/enabler]<br>**Cross-departmental communication:**<br>- FEEBACK PORTAL: *Communication with other depts* [barrier/enabler]
**Culture**	Norms, values, and basic assumptions of a given organisation.	- Service provider interviews: providers will be asked what improvements are needed and what is working well with regards to the care provided at their service, as well as how well-equipped they feel to provide care to Aboriginal parents.<br>- Data from service provider interviews will be discussed in SIT meetings and will inform implementation activities.<br><br>Questions relating to subconstructs:<br>**Shared values:**<br>- ORA: *People who work here want to implement this change.*<br>- FEEDBACK PORTAL: *People who work here share a commitment to improve support for Aboriginal and Torres Strait Islander families.*<br>**Prevalent Sentiments:** KAP attitude scores<br>**Learning culture:** Assessed through uptake of HPNF training.<br>- FEEDBACK PORTAL: *People who work here are interested in learning and professional development.*<br><br>- Responses to these questions will be discussed in SIT meetings and will guide implementation activities.

**Implementation climate**	The absorptive capacity for change, shared receptivity of involved individuals to an intervention and the extent to which use of that intervention will be rewarded, supported, and expected within their organization.	- Questions in the ORIC, KAP and BETICI surveys will assess the implementation climate prior to implementation.<br><br>Questions relating to subconstructs:<br>**Tension for change:**<br>- Service providers will be asked in interviews what is and isn't working, and what could improve in the care provided to parents.<br>- The ORIC assessment will be conducted prior to implementation to assess the degree to which stakeholders perceive the need for change to occur.<br>**Compatibility:**<br>- BETICI: *Trauma-informed approaches would be/are considered a burden on resources.*<br>- FEEDBACK PORTAL:<br>*I can easily work trauma-informed approaches into my role.*<br>*People who work here are open to changing their ways of working.*<br>**Relative Priority:**<br>- Service provider interviews will capture perceptions on the importance of adopting changes.<br>**Learning climate:**<br>- Assessed through the level of support given to staff to engage in training (recorded in SIT journal)
**Readiness for Implementation**	Tangible and immediate indicators of organisational commitment to its decision to implement an intervention.	- Overall readiness for implementation will be assessed through the ORIC and BETICI surveys in the pre-implementation period.<br><br>Questions relating to subconstructs:<br>**Leadership engagement:**<br>- BETICI: *Management would/do support trauma-informed training for all staff.*<br>- FEEDBACK PORTAL: *I have the support I need from my supervisors to use a trauma-informed approach.*<br>**Available resources:**<br>- Resources available for implementation will be tracked by the SIT and recorded in SIT implementation journal.<br>- FEEDBACK PORTAL: *The number of staff we have* [barrier/enabler]

		**Access to Knowledge and Information:**<br>- BETICI:<br>*There is a lack of resources and information to learn about trauma-informed care.*<br>*There is local, context-specific training available about trauma-informed approaches*
**Leadership Qualities**	Descriptions of organization leaders or management that are not specific to the intervention or its implementation.	- FEEDBACK PORTAL: Senior leaders' commitment to overall service quality [barrier/enabler]
**Characteristics of individuals**		
**Knowledge and Beliefs about the Intervention**	Individuals' attitudes toward and value placed on the intervention as well as familiarity with facts, truths, and principles related to the intervention.	- KAP survey will explore knowledge and attitudes about trauma-informed care.
**Self-efficacy**	Individuals' belief in their own capabilities to execute courses of action to achieve implementation goals.	- ORIC: *People who work here feel confident they can handle the challenges that might arise in implementing this change.*<br>- FEEDBACK PORTAL: *I am confident using the strategies I learned in training*
**Individual Stage of Change**	Characterisation of the phase an individual is in, as they progress toward skilled, enthusiastic, and sustained use of the intervention.	- KAP survey questions will assess the stage of change through tracking scores before, immediately after and 3months post-training.<br>- FEEDBACK PORTAL:<br>*In the past 2 weeks, have you applied any knowledge or skills gained through the training?*<br>*Please describe how you have applied new knowledge or skills from training.*<br>*I am confident using the strategies I learned in training*

**Other Personal attributes**	A broad construct to include other personal traits such as tolerance of ambiguity, intellectual ability, motivation, values, competence, capacity, and learning style.	Questions relating to sub-constructs:<br>- **Busy:**<br>FEEDBACK PORTAL: *The amount of time I have* [barrier/enabler]<br>- **Descriptive information:** Demographic details will be collected through service provider interviews. When completing KAP and feedback portal, service providers will be asked to indicate their role, their experience in that role and the duration of employment within the organisation and the level of training completed.<br>- **Learning capacity:**<br>BETICI: *I am highly motivated to learn more about trauma-informed approaches.*<br>Attendance at information sessions and subsequent rate of registrations to participate in training.<br>FEEDBACK PORTAL: *My interest in learning and professional development* [enabler/barrier]
**Process**		
**Planning**	The degree to which a scheme or method of behavior and tasks for implementing an intervention are developed in advance and the quality of those schemes or methods.	- Participatory action research projects are highly iterative, open-ended, and unpredictable in nature. Rather than adhering to a set research design and methodology, PAR draws on collaboration, and context and responds to developments and address complex systems barriers and processes as they arise throughout the process (Cornish et al, 2023).
**Engaging**	Attracting and involving appropriate individuals in the implementation and use of the intervention through a combined strategy of social marketing, education, role modeling, training, and other similar activities.	- Engagement will be encouraged through a series of information sessions in the pre-implementation period and will be assessed through attendance rates and rates of subsequent registrations to training sessions.<br>- Training sessions will be promoted widely throughout relevant health services via flyers, displays, emails, and newsletter articles.<br>- Implementation strategies from training will be promoted through service provider resources including posters, desktop materials, magnets, postcards, wall guides.<br><br>Questions relating to subconstructs:<br>- **Formally Appointed Internal Implementation Leaders:**

		- **Champions:** A self-selected group of project champions will encourage colleagues to engagement with implementation activities. Training will be piloted with this group and feedback will drive adaptations for the wider group of participants. The champions will also inform the SIT of barriers and enablers to implementation to guide activities.<br>- **External Change Agents:** service providers external to the main implementation site will be interviewed to compare responses with those of internal staff, which will guide training content and delivery. These agents will take an active role in pre-implementation workshop 5 and will inform implementation activities.
**Executing**	Carrying out or accomplishing the implementation according to plan.<br>Executing is only assessed during or post implementation because it is determined by how well they executed the implementation plan. If executing has not gone according to plan, (double) code to the appropriate CFIR construct that explains why the plan did not work.	- A mixed-methods evaluation will be conducted to assess the reach, effectiveness, adoption, implementation, and maintenance.<br>- SIT implementation journaling will highlight implementation barriers, adherence and fidelity throughout the implementation period and solutions will be workshopped with HPNF action group and in Workshop 5<br>- Data from post-implementation service provider interviews will highlight implementation fidelity
**Reflecting & Evaluating**	Quantitative and qualitative feedback about the progress and quality of implementation accompanied with regular personal and team debriefing about progress and experience.	- Service providers will be encouraged to complete a feedback survey in which they can provide feedback and reflections on what is helping and hindering implementation as well as general comments.<br>- SIT will complete at least fortnightly journalling to enable reflection on implementation activities throughout the process.<br>- Post-implementation interviews will enable evaluation and reflection by service providers.

ORA= Organisational Readiness Assessment, SIT = Site Implementation Team, BETICI= Barriers and Enablers to Trauma-Informed Care Implementation, KAP = Knowledge Attitudes Practice survey
