# Supplementary material for: Healing the Past by Nurturing the Future: trauma-aware, healing-informed care to improve support for Aboriginal and Torres Strait Islander families – implementation and evaluation study protocol
Source: BMJ Open. 2024 Jul 2;14(7):e085555. doi: 10.1136/bmjopen-2024-085555 (PMC11227778; doi:10.1136/bmjopen-2024-085555)
Supplement: online supplemental file 2 [file bmjopen-2024-085555-s002.pdf]

Supplementary file 2

RE-AIM evaluation plan

Evaluation Question	Indicator	Method
REACH		
Do service providers attend training?	- Number of service providers attending training	- Implementation log
Have parent resources been distributed?	- Number of resources distributed	- Implementation log
EFFECTIVENESS		
Are parent resources perceived to be useful?	- Feedback from parents about usefulness of resources	- Post-implementation interviews with 15-20 parents
Is the provision of trauma-aware care acceptable and appropriate for parents?	- Feedback from parents about acceptability and appropriateness of trauma-aware care	- Post-implementation interviews with 15-20 parents
Does implementation of the culturally safe, trauma-aware approach to care result in changes in child and parent health outcomes?	- Number of child protection notifications<br>- Number of antenatal visits attended<br>- Gestational age<br>- Baby birthweight<br>- Number of neonatal special care admissions<br>- Number of babies breastfeeding at hospital discharge<br>- Number of babies receiving 1-month vaccination	- Analysis of trends in secondary linked administrative data collected for a total period of 6 years including 2 years pre-implementation, approximately 1-2 years of implementation roll out, and 2 years post-implementation
Does implementation of the culturally safe, trauma-aware approach to care result in changes in parent health and wellbeing outcomes?	- Decreased symptoms of complex trauma<br>- Increased parenting self-efficacy score<br>- Decreased psychological distress score	- ACTSQ, TOPSE, K5 surveys 2-3-months pre- and post-implementation, with 8-10 parents who birthed at the implementation site in past 3-9-months
Does the training increase service providers' knowledge, attitudes and practice in relation to trauma?	- Increase in knowledge and change in attitudes	- KAP survey of service providers pre- and post-training and 3-months post-training
ADOPTION		

Evaluation Question	Indicator	Method
Do service providers provide trauma-aware care to parents as intended?	- Feedback and reflections from service providers on their service delivery for Aboriginal parents who may be experiencing complex trauma<br>- Changes to organisational processes and procedures	- Post-implementation interviews with 15-20 service providers<br>- Policy document review
Are service managers and executive staff committed to implementing the change in service delivery?	- Management and executive level engagement with training and SIT activities	- SIT Journal<br>- REDCap questionnaire<br>- SP feedback portal<br>- Service provider interviews
IMPLEMENTATION		
Is the training useful for service providers?<br>Is the training feasible?	- Feedback from service providers on feasibility and usefulness of training	- Post-implementation interviews with 15-20 service providers
How does the support framework support parents?	- Reflections from SIT on how the framework supports parents	- SIT journal<br>- Post-implementation interviews with 15-20 service providers
What are the barriers and enablers that service providers face in delivering trauma-aware care?	- Reported barriers and enablers from service providers and SIT team	- Pre- and post-implementation interviews with 15-20 service providers<br>- BETICI questionnaire<br>- Implementation log<br>- SIT journal<br>- SP feedback portal
What are the barriers and enablers to implementation, and factors that may affect implementation in other settings?	- How well employees at the organization feel they can implement change in processes required by the proposed intervention	- All implementation site staff invited to complete adapted ORIC survey pre-implementation
What is the cost of implementing trauma-aware care compared to usual care?	- Time data (as below)<br>- Salary scales<br>- Costs of developing training and resources	- Implementation log<br>- Cost evaluation
What is the time commitment to implement trauma-aware care compared to usual care?	- Time spent by staff on training, refresher training.	- Implementation log

Evaluation Question	Indicator	Method
	- Time spent on mentoring/supervision<br>- Time spent on reflective practice	
Was the intervention delivered as intended?	- Self-report or observations of delivery by service providers	- Post-implementation interviews with 15-20 service providers<br>- Implementation log
MAINTENANCE		
What is the impact on policy and practice?	- Reported or documented changes to policy and practice	- Post-implementation interviews with 15-20 service providers<br>- Policy document review

SIT=Site Implementation Team, KAP=Knowledge, Attitudes, Practice survey, ORIC=Organizational Readiness for Implementing Change Survey, TOPSE= Tool to Measure Parenting Self-Efficacy, BETICI= Barriers and Enablers to Trauma-Informed Care Implementation, K5= Kessler Psychological Distress Scale
