# Supplementary material for: Healing the Past by Nurturing the Future: trauma-aware, healing-informed care to improve support for Aboriginal and Torres Strait Islander families – implementation and evaluation study protocol
Source: BMJ Open. 2024 Jul 2;14(7):e085555. doi: 10.1136/bmjopen-2024-085555 (PMC11227778; doi:10.1136/bmjopen-2024-085555)
Supplement: Supplementary file 3 [file bmjopen-2024-085555.reviewer_comments.pdf]

PEER REVIEW HISTORY

BMJ Open publishes all reviews undertaken for accepted manuscripts. Reviewers are asked to complete a checklist review form (**http://bmjopen.bmj.com/site/about/resources/checklist.pdf**) and are provided with free text boxes to elaborate on their assessment. These free text comments are reproduced below.

ARTICLE DETAILS

**TITLE (PROVISIONAL)**	Healing the Past by Nurturing the Future: Trauma-aware, Healing-informed Care to Improve Support for Aboriginal and Torres Strait Islander Families: Implementation and Evaluation Study Protocol
**AUTHORS**	Jones, Kimberley; Henderson, Helen; Bright, Tess; Segal, Leonie; Mauerhofer, Olivia; Lake, Katherine; Julian, Rebakah; Duncan, Jhodie; Raymond, Anita; Jones, Amanda; Cameron, Danielle; Fergie, Doseena; Andrews, Shawana; Stewart, Skye; Atkinson, Caroline; Elliot, Alison; Crawford, Belinda; Mohammed, Janine; Bundle, Gina; Hirvonen, Tanja; Gnanamanickam, Emmanuel; Davis, E; Gee, Graham; Herrman, Helen; Fisher, Jane; Lovett, Raymond; Campbell, Sandy; Forster, Della; Clark, Yvonne; Atkinson, Judith; Marriott, Rhonda; Chamberlain, Catherine

VERSION 1 – REVIEW

**REVIEWER**	Coombes, Julieann<br>The George Institute
**REVIEW RETURNED**	08-Apr-2024

**GENERAL COMMENTS**	This is a very well written manuscript and 1 that is worthy of sharing.

**REVIEWER**	Secombe, Paul J<br>Alice Springs Hospital, ICU
**REVIEW RETURNED**	01-May-2024

**GENERAL COMMENTS**	Thankyou for asking me to review this implementation and evaluation protocol for the HPNF project aimed at improving support for Aboriginal and Torres Strait Islander families experiencing complex trauma<br><br>The research questions that the HPNF program aims to answer, include<br>i) exploring the experiences of Aboriginal and Torres Strait Islander parents receiving trauma-aware, healing-informed perinatal care,<br>ii) identifying the barriers and enablers to implementation, assessing the cost-effectiveness of the program, and<br>iii) evaluating the impact on health and socio-emotional outcomes for parents and their infants<br><br>It is mostly well written and covers an important area. I have no major concerns or comments, only a number of minor points.<br><br>Minor concerns

	The first paragraph of the article attempts to link mortality rates, rates of OOHC, and intergenerational trauma with perinatal care. While probably associated, the mechanism(s) by which improved perinatal care flows through to the first three is not made, and could be made more explicit.<br><br>Trauma carries multiple meanings depending on context, and can be further subdivided by physical trauma and psychological trauma (and it is presumably the latter with which this study deals). It would help to make this more explicit in the opening paragraphs, perhaps drawing on the DSMV definition of a "traumatic event"<br><br>The authors need to check the referencing - reference 1 is doubled up as reference 32, reference 33 is incomplete (it has been published in the Journal of Indigenous Well Being), and refers 34 is available on line (Microsoft Word - Healing the Past by Nurturing the Future W1 Report final draft v5 (latrobe.edu.au)which is not indicated in the reference. I am not sure whether there are other referencing errors.<br><br>Do the authors genuinely believe that re-traumatisation can be completely avoided (p. 10 line 198), or is this an aspirational goal, and what is really being sought is risk mitigation - reducing the risk of re-traumatisation to a value as low as possible?<br><br>Why has another comparator LGA not yet been chosen? One would have thought this is exactly what a study protocol should be specifying in advance. (p. 14 line 307 - 310)<br><br>Do the authors already know the number of staff to whom survey invitations will be sent (p 16 lines 355 - 360)? Presumably the number of email invitations sent out will form the denominator for the response rate? An estimate in the protocol would be useful.

VERSION 1 – AUTHOR RESPONSE

Reviewer: 1
This is a very well written manuscript and 1 that is worthy of sharing.
Thank you, we are glad you think so.
Reviewer: 2
Thankyou for asking me to review this implementation and evaluation protocol for the HPNF project aimed at improving support for Aboriginal and Torres Strait Islander families experiencing complex trauma
The research questions that the HPNF program aims to answer, include
i) exploring the experiences of Aboriginal and Torres Strait Islander parents receiving trauma-aware, healing-informed perinatal care,
ii) identifying the barriers and enablers to implementation, assessing the cost-effectiveness of the program, and
iii) evaluating the impact on health and socio-emotional outcomes for parents and their infants

It is mostly well written and covers an important area. I have no major concerns or comments, only a number of minor points.
Thank you.

Minor concerns

The first paragraph of the article attempts to link mortality rates, rates of OOHC, and intergenerational trauma with perinatal care. While probably associated, the mechanism(s) by which improved perinatal care flows through to the first three is not made, and could be made more explicit.
Thank you for highlighting this point. We agree our statements were poorly linked and have now amended this paragraph to provide a more coherent explanation on how perinatal care is associated with these outcomes.

Trauma carries multiple meanings depending on context, and can be further subdivided by physical trauma and psychological trauma (and it is presumably the latter with which this study deals). It would help to make this more explicit in the opening paragraphs, perhaps drawing on the DSMV definition of a “traumatic event”
Excellent point we have addressed this in the second paragraph.

The authors need to check the referencing - reference 1 is doubled up as reference 32, reference 33 is incomplete (it has been published in the Journal of Indigenous Well Being), and refers 34 is available on line (Microsoft Word - Healing the Past by Nurturing the Future W1 Report final draft v5 (latrobe.edu.au)which is not indicated in the reference. I am not sure whether there are other referencing errors.
Thank you for highlighting these errors. We have now done a review of all references and corrected any errors.

Do the authors genuinely believe that re-traumatisation can be completely avoided (p. 10 line 198), or is this an aspirational goal, and what is really being sought is risk mitigation - reducing the risk of re-traumatisation to a value as low as possible?
Fair point – you are correct that this is aspirational rather than realistic. We have now amended this sentence to reflect that we are seeking to minimise the risk of re-traumatisation.

Why has another comparator LGA not yet been chosen? One would have thought this is exactly what a study protocol should be specifying in advance. (p. 14 line 307 - 310)
Good question. We are collecting data for all LGAs so we can determine which LGA aligns most closely with the implementation site in terms of a range of variables data for which are otherwise unavailable – this was recommended by the data linkage authority as the best way to ensure a well-matched comparator rather than limiting data linkage with one site only to discover it was a poor match across these important variables.

Do the authors already know the number of staff to whom survey invitations will be sent (p 16 lines 355 - 360)? Presumably the number of email invitations sent out will form the denominator for the response rate? An estimate in the protocol would be useful.
We have now added an estimate for this sample.

VERSION 2 – REVIEW

**REVIEWER**	Secombe, Paul J<br>Alice Springs Hospital, ICU
**REVIEW RETURNED**	30-May-2024

**GENERAL COMMENTS**	Thank you .. the responses are more than adequate
